# Sulfoquinovose degradation by cow rumen microbiota

**DOI:** 10.1093/ismejo/wrag069

**Published:** 2026-03-27

**Authors:** Julia Krasenbrink, Song-Can Chen, Tomohisa Sebastian Tanabe, Hüseyin Sarikeçe, Pleun Meurs, Sabrina Borusak, Rahul Samrat, Guoqing Guan, Clara Priemer, Jay Osvatic, Joana Séneca, Bela Hausmann, Daan R Speth, Evelyne Selberherr, Wolfgang Wanek, David Schleheck, Marc Mussmann, Alexander Loy

**Affiliations:** Division of Microbial Ecology, Centre for Microbiology and Environmental Systems Science, University of Vienna, A-1030 Vienna, Austria; Doctoral School in Microbiology and Environmental Science, University of Vienna, A-1030 Vienna, Austria; Division of Microbial Ecology, Centre for Microbiology and Environmental Systems Science, University of Vienna, A-1030 Vienna, Austria; State Key Laboratory of Soil Pollution Control and Safety, Zhejiang University, 310058 Hangzhou, China; MOE Key Laboratory of Environment Remediation and Ecological Health, College of Environmental and Resource Sciences, Zhejiang University, 310058 Hangzhou, China; Division of Microbial Ecology, Centre for Microbiology and Environmental Systems Science, University of Vienna, A-1030 Vienna, Austria; Division of Microbial Ecology, Centre for Microbiology and Environmental Systems Science, University of Vienna, A-1030 Vienna, Austria; Division of Microbial Ecology, Centre for Microbiology and Environmental Systems Science, University of Vienna, A-1030 Vienna, Austria; Department of Biology, University of Konstanz, D-78457 Konstanz, Germany; Konstanz Research School Chemical Biology, University of Konstanz, D-78457 Konstanz, Germany; Doctoral School in Microbiology and Environmental Science, University of Vienna, A-1030 Vienna, Austria; Centre for Microbiology and Environmental Systems Science, Division of Terrestrial Ecosystem Research, University of Vienna, A-1030 Vienna, Austria; Division of Microbial Ecology, Centre for Microbiology and Environmental Systems Science, University of Vienna, A-1030 Vienna, Austria; Doctoral School in Microbiology and Environmental Science, University of Vienna, A-1030 Vienna, Austria; Doctoral School in Microbiology and Environmental Science, University of Vienna, A-1030 Vienna, Austria; Centre for Microbiology and Environmental Systems Science, Division of Terrestrial Ecosystem Research, University of Vienna, A-1030 Vienna, Austria; Joint Microbiome Facility of the Medical University of Vienna and the University of Vienna, A-1030 Vienna, Austria; Division of Clinical Microbiology, Department of Laboratory Medicine, Medical University of Vienna, A-1090 Vienna, Austria; Division of Microbial Ecology, Centre for Microbiology and Environmental Systems Science, University of Vienna, A-1030 Vienna, Austria; Joint Microbiome Facility of the Medical University of Vienna and the University of Vienna, A-1030 Vienna, Austria; Division of Clinical Microbiology, Department of Laboratory Medicine, Medical University of Vienna, A-1090 Vienna, Austria; Joint Microbiome Facility of the Medical University of Vienna and the University of Vienna, A-1030 Vienna, Austria; Division of Clinical Microbiology, Department of Laboratory Medicine, Medical University of Vienna, A-1090 Vienna, Austria; Division of Microbial Ecology, Centre for Microbiology and Environmental Systems Science, University of Vienna, A-1030 Vienna, Austria; Unit of Food Microbiology, Institute of Food Safety, Food Technology and Veterinary Public Health, University of Veterinary Medicine, A-1210 Vienna, Austria; Centre for Microbiology and Environmental Systems Science, Division of Terrestrial Ecosystem Research, University of Vienna, A-1030 Vienna, Austria; Department of Biology, University of Konstanz, D-78457 Konstanz, Germany; Konstanz Research School Chemical Biology, University of Konstanz, D-78457 Konstanz, Germany; Division of Microbial Ecology, Centre for Microbiology and Environmental Systems Science, University of Vienna, A-1030 Vienna, Austria; Division of Microbial Ecology, Centre for Microbiology and Environmental Systems Science, University of Vienna, A-1030 Vienna, Austria; Joint Microbiome Facility of the Medical University of Vienna and the University of Vienna, A-1030 Vienna, Austria

**Keywords:** sulfoquinovose, sulfur cycle, organosulfur, sulfolipid, gut microbiome, rumen, functional gene, sulfoquinovosidase

## Abstract

Sulfoquinovose, a sulfonated sugar derived from the thylakoid membrane lipid sulfoquinovosyl diacylglycerol, is abundant in photosynthetic organisms and plays a key role in global sulfur cycling. Its degradation in nature is mediated by specialized bacteria, many of which rely on the enzyme sulfoquinovosidase (YihQ) to release sulfoquinovose from sulfoquinovosyl (diacyl)glycerol. Despite its ecological importance, the diversity and functional roles of sulfoquinovose-degrading microorganisms remain poorly characterized in natural environments. Here, we developed a *yihQ*-targeted amplicon sequencing approach to investigate the richness and distribution of SQ-degrading bacteria across selected environments. We revealed high richness of *yihQ*-containing microorganisms in the analyzed cow rumen samples, far exceeding that observed in human and mouse gut microbiomes, suggesting an important role of sulfoquinovose metabolism in ruminant digestion. Anoxic microcosm experiments with sulfoquinovose-amended rumen fluid revealed cooperative microbial degradation of sulfoquinovose to sulfide via isethionate cross-feeding. Amplicon sequencing and genome-resolved metagenomics and metatranscriptomics identified yet undescribed and uncultured sulfoquinovose-degrading taxa. Members of *Caproiciproducens* (*Acutalibacteraceae*), *Candidatus* Limivicinus (*Oscillospiraceae*), and *Sphaerochaetaceae* transcribed the isethionate-producing sulfo-transketolase pathway, whereas isethionate was likely respired by a *Candidatus* Mailhella bacterium (*Desulfovibrionaceae*). This study presents a functional gene-based assay for tracking environmental *yihQ* richness, highlights sulfoquinovose degradation as a central metabolic process in the cow rumen, describes previously unknown sulfoquinovose-metabolizing bacteria, and advances understanding of sulfur physiology in complex microbial communities.

## Introduction

Sulfoquinovose (SQ; 6-deoxy-6-sulfo-D-glucose) is a sulfonated hexose of global importance [1, 2]. Approximately 10 billion tons of SQ are produced globally per year, comparable to the amino acids methionine and cysteine. SQ is an integral component of the biogeochemical sulfur cycle and the human and animal diet [1, 2]. Free SQ appears to be rare in nature and is primarily present as the head group of the sulfolipid sulfoquinovosyl diacylglycerol (SQDG) in the biomass of photosynthetic organisms such as plants, algae, and cyanobacteria [2–6]. SQDG resides in the thylakoid membrane, where it supports the activity and structural integrity of photosystem II [2, 7, 8].

To date, several pathways for SQ degradation have been identified in bacteria. Some pathways require inter-species transfer of organosulfonates for complete SQ mineralization to sulfate or hydrogen sulfide [9–15]. In contrast, the SQ monooxygenase pathway in *Agrobacterium tumefaciens* and the *Roseobacter* clade and the SQ dioxygenase pathway in *Marinomonas ushuaiensis* aerobically cleave the C-S bond of SQ and directly catabolize SQ to sulfite and a deoxy-hexose [16–18]. Because SQDG is the main natural source of SQ, all known pathways require the enzymatic release of SQ from SQDG or sulfoquinovosyl glycerol [4, 19]. In five of the six physiologically and biochemically characterized SQ degradation pathways, this cleavage is catalyzed by sulfoquinovosidases of the glycoside hydrolase family 31 [4, 10, 11, 13, 14, 16, 17, 19–21]. These include YihQ in *Escherichia coli* [10], *Akalicoccus urumqiensis* (formerly *Bacillus urumqiensis*) [14], *Clostridium* sp. [14], and *Novosphingobium aromaticivorans* [14], PpSQ1_00094 in *Pseudomonas putida* [11], SftG in *Priestia aryabhattai* (formerly *Bacillus aryabhattai*) [13], SqvC in *Priestia megaterium* (formerly *Bacillus megaterium*) [21], and SmoI in *A. tumefaciens* [16]. These sulfoquinovosidases have conserved structural and functional features and form a monophyletic orthologous group in the glycoside hydrolase family 31 tree [4, 19]. We therefore refer to members of this orthologous group as YihQ sulfoquinovosidases. YihQ from *E. coli* and *A. tumefaciens* hydrolyzed both SQDG and sulfoquinovosyl glycerol in vitro [4, 19]. *E. coli* cultures supplemented with sulfoquinovosyl glycerol or glucose grew to the same optical density. In these growth experiments sulfoquinovosyl glycerol was metabolized faster than glycerol and free sulfoquinovose, but later than glucose. Growth rates of cultures with sulfoquinovosyl glycerol were also higher than those of cultures grown with SQ or glycerol alone, indicating that sulfoquinovosyl glycerol is the preferred substrate for *E. coli* [19].

YihQ genes are widespread among bacteria that encode SQ-degradation pathways. Some SQ-degrading bacteria, such as *Roseobacter* spp., *Arthrobacter* spp., and *Marinomonas* sp., lack a *yihQ* homolog [17, 18, 22]. However, a new family of NAD^+^-dependent sulfoquinovosidases was recently discovered and is widely distributed in bacteria that were previously thought to lack a sulfoquinovosidase [23].

SQDG and SQ are integral parts of the sulfur cycle, yet the diversity of SQ-degrading microorganisms across different environments remains largely unexplored, with studies to date limited to human and mouse gut microbiomes and seawater [18, 24, 25]. The presence of *yihQ* homologs in five of the six SQ degradation pathways suggests that this gene represents a suitable functional marker for many SQDG/SQ-degrading microorganisms. To this end, we developed two *yihQ*-targeted degenerate primer sets and evaluated their use for analyzing the *yihQ* sequence diversity in selected environmental (soil, marine sediments) and gut (human, cow, and mouse) samples. Furthermore, the *yihQ* amplicon sequencing approach was combined with 16S rRNA gene amplicon sequencing, metagenomics, metatranscriptomics, and metabolite analysis to study SQ degradation and the involved microorganisms and pathways in cow rumen fluid microcosms. We show that the SQ-degrading community in the cow rumen differs from those in the mouse and human gut by comprising several hundred distinct *yihQ* sequences, consisting of undescribed species, and predominantly utilising the sulfo-transketolase pathway for SQ degradation to isethionate.

## Materials and methods

Supplementary Materials and Methods provide further details on the methods described below.

### Design of *yihQ*-targeted PCR assays

A YihQ reference database was created using sequences of biochemically validated YihQ sulfoquinovosidases and homologs of functionally different enzymes of the glycoside hydrolase family 31 as queries for BLAST against the KEGG prokaryotes database [26] (Table S1). An alignment of representative sequences was used to calculate a maximum-likelihood YihQ tree (Fig. S1) and for the design of degenerate *yihQ*-targeted primers. Genomic DNA of the *yihQ*-encoding strains *E. coli* K12, *Agathobacter rectalis* A1–86, and *Enterocloster clostridioformis* YL32 were used as positive controls for PCR. DNA of *Segatella copri* DSM 18205 and ultrapure water served as non-target control and negative control, respectively. Primer pairs targeting different *yihQ* regions were initially tested using pure culture DNA for temperature gradient PCR and further optimized by touchdown PCR. The optimized PCR program (Table S2) was subsequently tested with DNA from several environmental and intestinal samples.

### Sampling and ethics

Marine coastal surface sediment samples (0–3 cm depth) were collected from Canada (Vancouver: 49°17′57.7”N, 123°10′34.6”W), Italy (Fetovia: 42°43′57.2”N, 10°9′15.6″E), Mauritania (Dakhlet Nouadhibou: 20°36′52.7”N, 16°30′35.9”W), and Australia (Casuarina Beach: 12°21′39.96”S, 130°51′56.7″E). A soil sample was collected from the acidic peatland Schlöppnerbrunnen II, Germany (50°07′54.8″N, 11°52′51.8″E, 10–20 cm depth), as previously described [27].

A human fecal sample mix was created by pooling freshly collected feces from five adult volunteers. Sampling and microbiota analyses of human feces was approved by the ethics commission of the University of Vienna (reference numbers 00161 and 00714). A mouse gut content sample mix was created by pooling small intestinal and cecal contents from eight conventional C57BL/6 wild-type mice (four females, four males, aged 8–10 weeks) as described previously [25]. Mice were housed under specific-pathogen-free conditions in compliance with the Federation of European Laboratory Animal Science Associations guidelines, with *ad libitum* access to a standard chow diet (Ssniff V1534, Ssniff, Germany) and water. Rumen fluid was collected at an International Featured Standard-Food certified slaughterhouse in Lower Austria. Cows originated from the same farm, were fed hay and grass silage *ad libitum*, with a maximum of 0.5 kg/day of commercial concentrate (KuhKorn PLUS Energie; Garant-Tiernahrung GmbH, Austria), and had free access to water, which is common practice in the region. The mechanical isolation procedure and gastrointestinal tract removal were performed directly post mortem (15–30 minutes after stunning and exsanguination) by licensed veterinarians following standard operating protocols to minimize contamination and preserve microbial composition [28]. A volume of 50 ml of rumen fluid was obtained from each rumen under controlled hygienic conditions. Samples were immediately transported to the laboratory and processed. During the first collection, a sample was obtained from one cow and used for quantifying SQDG and for *yihQ*-amplicon sequencing alongside with other environmental samples. During the second collection, one sample each was obtained from three cows and was used for the rumen fluid microcosm experiment. As the mouse gut and rumen fluid samples were obtained post-mortem, no ethical approval was required for this study.

### Rumen fluid microcosms

Anoxic rumen fluid microcosms supplemented with 10 mM SQ (MCAT GmbH, Germany) were used to investigate SQ metabolism by the cow gut microbiome (Supplementary Materials & Methods). Microcosms were subsampled over 168 hours for metabolite quantification and microbial community analyses.

### Pure culture growth tests

*Caproiciproducens* strain KNHs216 (DSM 100560), which has been isolated from forest soil seeded microcosms evolved on switchgrass, was obtained from the DSMZ-German Collection of Microorganisms and Cell Cultures. Anaerobic growth tests with and without 10 mM SQ were performed in a microwell plate reader and in Hungate tubes for metabolite quantification (Supplementary Materials & Methods). Growth was quantified by measuring optical density at 600 nm.

### Metabolite analysis

Ultra-high-performance reverse phase liquid chromatography combined with mass spectrometry was used to quantify and identify all molecular species of SQDG in rumen fluid (Supplementary Materials & Methods). Sulfide was quantified colorimetrically as previously described [29]. Formate, succinate, acetate, propionate, butyrate, valerate, and lactate were quantified by capillary electrophoresis. SQ and its metabolites isethionate, 2,3-dihydroxypropane-1-sulfonate (DHPS), 3-hydroxypropane-1-sulfonate, 3-sulfolactate, and 3-sulfopropionate were quantified by liquid chromatography–mass spectrometry as described previously (Supplementary Materials & Methods) [30]. Ion chromatography was used to quantify SQ and isethionate in culture supernatants of *Caproiciproducens* strain KNHs216 cultures (Supplementary Materials & Methods).

### 16S rRNA gene and *yihQ* amplicon sequencing and analyses

Genomic DNA from environmental, intestinal, and rumen fluid microcosm samples was isolated using the DNeasy PowerSoil Pro Kit (Qiagen, Austria). Amplicon sequencing on a MiSeq System (V3, 600 cycles, Illumina, USA) was conducted by the Joint Microbiome Facility (University of Vienna, Medical University of Vienna) under project IDs JMF-2310-13 and JMF-2303-02. DNA from each environmental sample was sequenced in triplicates within the same sequencing run. A two-step PCR method [31, 32] and primer pairs YIHQa (YIHQ1201Fa/YIHQ1526Ra) and YIHQb (YIHQ1201Fb/YIHQ1526Rb) were used to amplify and barcode an ~340 bp region of the *yihQ* gene (Fig. S2, Table S2). Samples from the rumen fluid microcosm experiment were analyzed by amplicon sequencing with primer pair YIHQb and with 16S rRNA gene-targeted primers for bacteria and archaea [31, 32]. Amplicon sequence variants (ASVs) were inferred using DADA2 [33]. FASTQ reads 1 and 2 were trimmed at 220 nt and 150 nt, respectively, with allowed expected errors of 2. 16S rRNA gene-ASVs were classified using DADA2 and the SILVA 16S rRNA sequence database (release 138.1) [34]. A workflow was established for analysis of sequences obtained with *yihQ*-targeted primers, including removal of non-*yihQ* sequences and phylogenetic placement in the YihQ reference tree (Fig. S3, Supplementary Materials & Methods). Verified *yihQ* ASV sequences were further clustered into operational taxonomic units (OTUs) using vsearch [35] with a 90% sequence identity threshold. Alpha diversity metrics (observed number of ASVs and OTUs, Shannon Index, and Simpson Index) were calculated for subsampled (100, 1000, and 10 000 reads) *yihQ* sequence libraries. Verified *yihQ* ASV sequences generated with the YIHQb amplicon sequencing assay were additionally classified using the GlobDB genome database (version r220) [36].

Significantly enriched 16S rRNA gene- and *yihQ-*OTUs/ASVs in SQ-supplemented rumen fluid microcosms were identified by DESeq2 (v1.38.3) as described previously [37]. Enriched ASVs/OTUs were taxonomically classified by BLAST against the NCBI 16S rRNA sequence database and the GlobDB database [36, 38].

### Metagenomics and genome analysis

Metagenomic sequencing was performed on a NovaSeq6000 (1/2 SP flow cell, 2 × 100 bp reads; Illumina, USA) with DNA extracts from SQ-amended (n = 2) and unamended control (n = 1) microcosms of rumen fluid, taken after 168 hours of incubation. Sequencing was conducted by the Joint Microbiome Facility (University of Vienna and Medical University of Vienna) under project ID JMF-2303-02. Metagenome-assembled genomes (MAGs) were dereplicated and taxonomically classified using GTDB-Tk [39]. Average amino acid identity (AAI) was calculated using the Enveomics Collection [40]. Whole-genome average nucleotide identity (ANI) was calculated using FastANI (version 1.33) [41]. Select genomes were annotated using the Prokaryotic Genome Annotation Pipeline of NCBI run as a container with Apptainer for reproducibility (pgap_2024-07-18.build7555.sif).

New Hidden Markov Models (HMMs) were generated for the detection of enzymes and transport proteins of sulfoquinovose, isethionate, and DHPS degradation pathways (Table S3). HMMs were integrated into HMSS2 [42], which was then used to identify sulfur metabolism genes in the recovered MAGs and GlobDB genomes (Supplementary Materials & Methods).

### Metatranscriptomics

Triplicate SQ-amended rumen fluid microcosms were sampled after 24 h and 120 h for RNA extraction and metatranscriptome sequencing on a NovaSeq 6000 (1/2 SP flowcell, 2x100bp reads; Illumina, USA), which was performed by the Joint Microbiome Facility (University of Vienna and Medical University of Vienna) under project ID JMF-2507-01 (Supplementary Materials & Methods). Transcriptome read libraries were aligned to the reference genomes using BBMap v39.26 (≥95% identity) and the “best” option. Counts of mapped reads for individual transcripts were obtained using SAMtools v1.21. [43]. The resulting counts were analyzed using DESeq2 [37] to determine differential expression between time points. However, the average number of reads per target genome at 24 h of incubation was too low (177–13493) for a reliable comparative analysis to the 120 h data (4207–2147207). Rank-abundance plots were used to visualize transcriptional patterns and highlight genome-wide transcription levels of genes associated with SQ and isethionate metabolism.

## Results and discussion

### Development of PCR assays for broad coverage of *yihQ* sequence diversity

Based on a curated YihQ sequence database and a YihQ reference tree (Fig. S1), we designed 10 degenerate primers that target different conserved regions in the *yihQ* alignment (Fig. S2, Table S4). Of the four tested primer combinations, only two primer pairs, YIHQa (YIHQ1201Fa/YIHQ1526Ra) and YIHQb (YIHQ1201Fb/YIHQ1526Rb) resulted in PCR products of the expected size with DNA templates of three *yihQ*-containing strains in the temperature gradient PCR tests. Primer pairs YIHQa and YIHQb were further tested in a touchdown PCR approach for enhanced specificity. The final protocol for both primer pairs used an initial annealing temperature of 63°C, which decreased by 1°C per cycle over 10 cycles, followed by 20 cycles at 53°C. Subsequent magnesium chloride concentration tests showed that 4 mM magnesium chloride in the PCR buffer consistently produced the strongest bands for the target sequences, whereas the negative and non-target DNA controls did not yield a visible PCR product (Table S2). Sanger sequencing of the PCR products confirmed specific amplification of the *yihQ* target gene in all three organisms.

The primers YIHQ1201Fb and YIHQ1526Rb were designed as shorter and less degenerate alternatives to YIHQ1201Fa and YIHQ1526Ra. Thus, primer pairs YIHQa and YIHQb amplify the same, ~340 bp region of *yihQ*, yet differ in degeneracy, predicted melting temperatures, and sequence coverage (Table S4), which may influence PCR amplification efficiency and specificity [44]. The YIHQa and YIHQb primer pairs match 81.8% and 89.8% of the *yihQ* sequences in the reference database without any mismatches, respectively. We thus applied and compared both primer pairs for amplification of DNA from various intestinal and environmental samples. These included pooled mouse gut content and human feces, which are known to harbor *yihQ*-containing bacteria [24, 25], and rumen fluid, soil, and diverse marine sediments. The latter are environments that have not yet been investigated for their SQ degradation capacity. Both PCR assays successfully amplified a single band of the expected size with DNA from all intestinal samples. PCRs with peat soil DNA were negative. PCRs with primer pair YIHQa produced faint PCR products with DNA from Canadian marine sediment, but no PCR products with DNA from Italian, Mauritanian, and Australian marine sediments. In contrast, PCRs with primer pair YIHQb generated bands for all marine sediment samples.

### Optimized *yihQ* amplicon sequencing reveals highest *yihQ* richness in cow rumen

We optimized the YIHQa and YIHQb PCR assays for amplicon sequencing on a MiSeq System (Illumina). Both primer pairs contained a generic 16 bp 5′-head sequence for two-step PCR barcoding [31, 32] (Table S2, Supplementary Materials & Methods). The optimized PCR protocol was applied to amplify DNA from the intestinal and environmental samples. Amplicons of the correct size were obtained with DNA from rumen fluid, mouse gut content, human feces, and Canadian sediment using both PCR assays. Additional amplicons with DNA from Mauritanian and Italian sediment were only obtained with the YIHQb assay, which suggests it has higher sensitivity and/or coverage. For technical replication, triplicate amplicons of each DNA sample that yielded a PCR product with both primer pairs were sequenced on a MiSeq System (Illumina).

**Figure 1:**
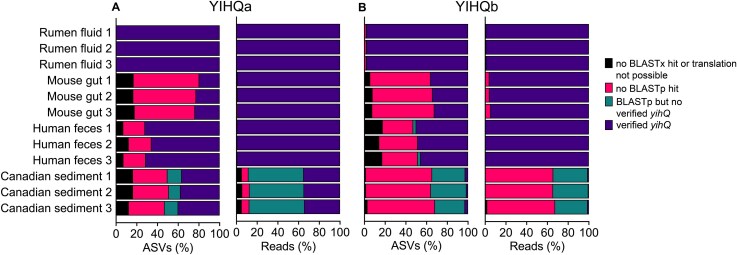
Specificity of the YIHQa and YIHQb amplicon sequencing assays is sample-dependent. Bar plots display the proportion of ASVs or reads (%) recovered from YIHQa (A) and YIHQb (B) amplicon sequencing assays that were verified as *yihQ* or excluded from further analysis (Fig. S3, Table S5). Each sample was sequenced in triplicates.

We established a computational workflow that allowed us to (i) assess the specificity of the YIHQa and YIHQb PCR assays and (ii) efficiently remove non-*yihQ* sequences from the dataset prior to further diversity analysis (Fig. S3). The workflow consisted of translating nucleic acid sequences into protein sequences, BLASTp against the YihQ reference database, and phylogenetic placement in the YihQ reference tree using the evolutionary placement algorithm in RAxML, which shows high accuracy for classification of functional gene sequences [45]. Both primer pairs amplified non-target sequences across all tested samples, but their relative abundance in the sequence libraries varied with sample type (Fig. 1A and B, Table S5). PCR specificity was highest for intestinal samples, particularly for rumen fluid samples, with >98% of reads and > 97% of ASVs verified as *yihQ* in both PCR assays. In contrast, less than 35% of reads and 40% of ASVs that were recovered with the YIHQa assay from the Canadian sediment sample were verified as *yihQ*. Hardly any verified *yihQ* reads and ASVs were recovered from sediment using the YIHQb assay. Overall, both YIHQa and YIHQb assays provided high specificity for *yihQ* sequence amplification from intestinal DNA samples but showed considerably lower reliability for the marine sediment DNA.

Sequences were further clustered into OTUs using a 90% similarity threshold, which has been used for other functional genes, such as *dsrA* and *dsrB* encoding dissimilatory sulfite reductase subunits, for approximate assignment into species-level groups [45]. Alpha diversity analysis at varying library sizes demonstrated high reproducibility among technical triplicates (Fig. S4, Table S6). Rumen fluid samples showed the highest *yihQ* richness with more than 1000 and 400 observed ASVs and OTUs, respectively. In contrast, mouse gut content and human feces samples, which were pooled from several individuals, had a considerably lower *yihQ* richness with only a few tens of observed ASVs and ~10 observed OTUs (Fig. S4). The low number of potential SQ-degrading species in the murine and human gut is consistent with prior reports [24, 25]. This difference in *yihQ* richness between the rumen and the human/mouse gut is considerable and needs to be confirmed in more comprehensive studies. The Canadian sediment sample had an intermediate *yihQ* richness with ~50 and 40 observed ASVs and OTUs, respectively. Accordingly, a library depth of only 100 sequences was sufficient to cover >98% of the expected ASV/OTU richness in the *yihQ* PCR product from the human and mouse gut samples, whereas hundred times more sequences were required to reach the same coverage for the rumen fluid sample (Fig. S4, Table S6).

**Figure 2:**
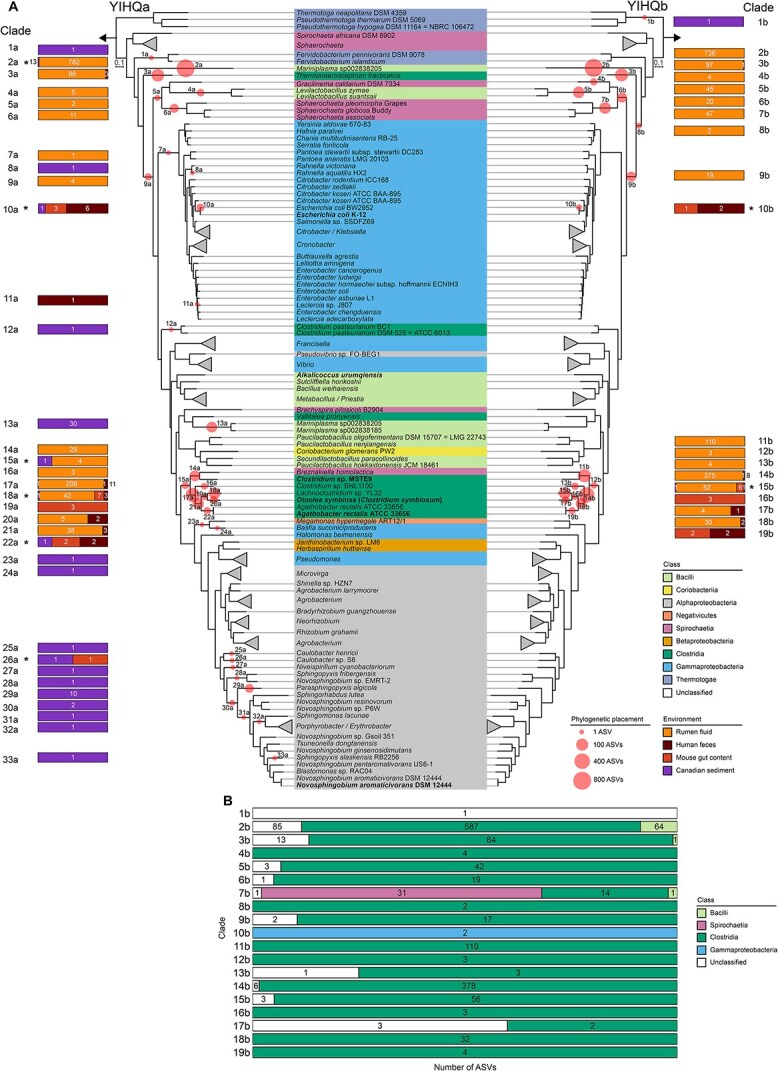
Phylogeny and taxonomic classification of environmental *yihQ* ASVs. Phylogeny of ASVs obtained from human feces, mouse gut content, rumen fluid, and marine sediment using the YIHQa assay (left tree) and the YIHQb assay (right tree) (A). For each ASV, a representative, translated sequence was added to the YihQ reference tree using the evolutionary placement algorithm of RAxML. The scale bar shows 10% sequence divergence. The class-level taxonomy of reference sequences is indicated in color. Sequence libraries were not rarefied before placement. Phylogenetic placement is indicated with red circles. Each circle represents an ASV clade. The circle size indicates the number of ASVs placed at that phylogenetic position. ASV clades are numbered. Bars correspond to ASV clades and show the percentage distribution of ASVs across environments, with numbers indicating the total ASV count, separately for each environment. Asterisks indicate identical ASVs found in different environments. In total, eight ASVs in YIHQa and two ASVs in YIHQb were detected across multiple environments (Table S7). Taxonomic composition of *yihQ* ASV clades generated in the YIHQb amplicon sequencing assay (B). Translated *yihQ* ASVs were queried against the GlobDB database using BLASTp. The top-scoring hit for each ASV was used for taxonomic assignment at the class-level. ASVs with no match or matches below 90% sequence identity were categorized as unclassified (white). Each horizontal bar represents an ASV clade, as defined in A, with individual segments showing the percentage distribution of ASVs to taxonomic classes within that clade. Numbers in bar plots refer to the absolute number of ASVs per class within one ASV clade. Taxonomic assignment of ASVs at the family and genus level is shown in Fig. S5 and Table S7.

The phylogenetic placement of the environmental *yihQ* sequences indicated that both assays recovered similar ASVs that were broadly distributed across the reference tree (Fig. 2). Most ASVs from rumen fluid (64% of 1220 ASVs from the YIHQa library; 47.5% of 1548 ASVs from the YIHQb library) clustered on a long branch corresponding to YihQ (protein BK011_07555 (AUD65555) of *Mariniplasma* sp002838205 strain MZ-QX (accession number SAMN05928844). Most ASVs from human feces (66% of 32 ASVs from the YIHQa library; 87% of 15 ASVs from the YIHQb library) and the mouse gut samples (71% of 17 ASVs from the YIHQa library; 92% of 13 ASVs from the YIHQb library) were affiliated with a *Clostridia* cluster of known SQ degraders, such as *Agathobacter rectalis* [24] and *Otoolea symbiosa* (formerly *Clostridium symbiosum*) [13]. Many rumen-derived ASVs (27% of 1220 ASVs from the YIHQa library; 37% of 1548 ASVs from the YIHQb library) were also affiliated with this cluster. Another subset of ASVs from human feces (19% of 32 ASVs from the YIHQa library; 13% of 15 ASVs from the YIHQb library) and the mouse gut (18% of 17 ASVs from the YIHQa library; 8% of 13 ASVs from the YIHQb library) clustered in the *Gammaproteobacteria*, which include the biochemically validated YihQ of *E. coli* [10]. Due to the low coverage of the YIHQb library (only two ASVs were recovered), the phylogenetic placements of ASVs from the two assays could not be compared for the Canadian marine sediment (Fig. 2). However, most marine ASVs from the YIHQa library were placed at a branch with a second YihQ gene copy (protein BK011_09290; AUD64050) of *Mariniplasma* sp002838205 strain MZ-QX (61% of 71 ASVs) or were affiliated with diverse YihQ sequences within the *Alphaproteobacteria* (27% of 71 ASVs). For refined taxonomic classification, all *yihQ* ASVs generated through the YIHQb assay were additionally matched against the GlobDB database, a comprehensive collection of bacterial and archaeal species genomes, using a conservative threshold of 90% YihQ sequence identity (Fig. S5, Table S7). Most ASVs were affiliated with *Oscillospiraceae, Lachnospiraceae*, and *Acutalibacteraceae* in the class *Clostridia* (Fig. S5).

The phylogenetic and sequence identity-based analysis provides an initial hypothesis for the taxonomic classification of unknown *yihQ*-containing microorganisms in environmental samples, which must be interpreted with caution. Some organisms, such as *Mariniplasma* sp002838205 strain MZ-QX and some *Candidatus* Limivicinus species, possess two copies of *yihQ*. Additionally, the phylogeny of YihQ does not always correspond to the taxonomy of the organisms (Fig. 2, Fig. S1). For example, numerous *yihQ* ASVs from cow rumen were assigned to the genus Ca. Limivicinus but form multiple phylogenetically distinct clades in the YihQ tree (Fig. 2, Fig. S5). These potential gene duplications and/or lateral transfers suggest that the evolutionary history of *yihQ* does not necessarily reflect the phylogenetic relationships of the host organisms. Similar to functional marker genes used for other microbial guilds, such as *aprBA, dsrAB*, and *amoA* [46–48], this complex evolutionary pattern presents challenges in taxonomic classification but does not preclude the use of *yihQ* as a marker for studying the environmental diversity of SQDG/SQ-degrading microorganisms.

In summary, the developed *yihQ*-selective PCR assays are currently not suitable for quantitative PCR due to the co-amplification of non-target sequences from environmental DNA. However, when applied to amplicon sequencing and combined with computational filtering of non-target reads, these assays enable specific assessment of *yihQ* richness in complex microbiome samples.

### Yet undescribed bacterial species degrade SQ to isethionate via the sulfo-transketolase pathway in cow rumen fluid microcosms

SQDG can be an abundant component of edible green plants and algae [2] and is thus a substrate for the intestinal microbiome. The identity and ecophysiology of intestinal SQDG/SQ-degrading bacteria has so far been only studied in humans [24] and mice [25]. Here, we performed rumen fluid microcosm experiments to investigate microbial SQ degradation in grass- and hay-fed cows, which presumably ingest substantial amounts of SQDG. The SQDG concentration in rumen fluid was 21 μg/g dry weight (Table S8). For comparison, the SQDG concentrations in plants range between 46 μg/g in garlic and 824 μg/g in spinach [49]. Data on SQDG content in grasses and hay are currently unavailable. SQDG molecules vary in their fatty acid composition, with differences in chain length and degree of saturation. These variations arise because plants produce a diversity of SQDG species depending on their physiology and environmental conditions [50]. In the rumen fluid, the SQDG species 18:3_16:0 represented ~70% of the total SQDG pool, which is similar to the SQDG species composition in green plants such as spinach, basil, and lettuce [51, 52].

**Figure 3:**
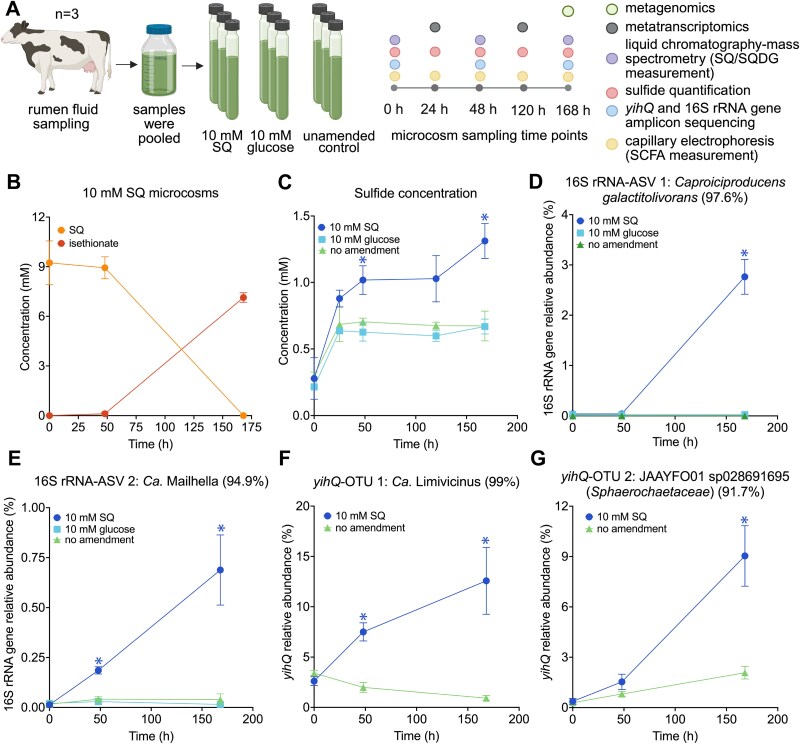
Yet uncultured *Caproiciproducens*, Ca. Limivicinus, Ca. Mailhella, and *Sphaerochaetaceae* species are involved in anaerobic degradation of sulfoquinovose to isethionate and sulfide in cow rumen microcosms. Overview of the rumen fluid microcosm experiment and the analytical approach (created with biorender.com) (A). Microcosms were amended with 10 mM SQ (n = 3) or 10 mM glucose (n = 3). Unamended microcosms (n = 3) served as controls. SQ (orange) degradation coincided with the production of ~7 mM isethionate (red) (B). SQ and isethionate were not detected in the unamended microcosms. Increase in sulfide concentration was significantly higher in SQ-amended microcosms (dark blue) compared to unamended controls (green) (C). Relative abundance dynamics of 16S rRNA gene-ASVs and *yihQ*-OTUs that were significantly enriched in SQ-amended microcosms compared to unamended controls (D-G). Sequence identity (%) to the next relative is shown in brackets. Data points and error bars represent the average of triplicate measurements and one standard deviation, respectively. Coloured asterisks show significant differences between the respective treatment group and the unamended control at individual time points. Significant differences were determined by ANOVA with Tukey’s posthoc test (C-E). Adjusted *P* values below 0.05 were considered significant. In G and F, significant differences were determined via DESeq2 analysis with *P* values of below 0.01 considered significant. SQ, sulfoquinovose; SQDG, sulfoquinovosyl diacylglycerol; SCFA, short-chain fatty acid; ASV, amplicon sequencing variant; OTU, operational taxonomic unit.

SQ concentrations decreased only slightly in SQ supplemented rumen fluid microcosms within the first 48 hours of anoxic incubation (Fig. 3A and B). However, SQ was completely metabolized after 168 h. No SQ was detected in the unamended control samples. SQDG or sulfoquinovosyl glycerol, rather than free SQ, likely serves as the natural source for SQ-degrading microorganisms. The SQ-degrading microorganisms in the rumen microcosms may have required upregulation of mechanisms for sensing and importing SQ, which could explain the prolonged lag phase prior to the onset of SQ degradation. SQ degradation coincided with the production of isethionate whereas other known organosulfonate SQ degradation products, such as DHPS or 3-sulfolactate [10–13], were not detected. Sulfide concentrations increased in all treatment groups but were significantly higher in SQ-amended microcosms compared to the unamended control (Fig. 3C). Temporal dynamics of acetate, lactate, propionate, and butyrate were not consistently different between SQ-amended microcosms and unamended controls (Fig. S6). However, the production of the known SQ fermentation products acetate and butyrate [12, 24, 25, 30] may have been masked by the high background concentrations of volatile fatty acids (Fig. S6), which is in agreement with previous studies [53, 54]. Despite the high richness of *yihQ*-containing microorganisms in the analyzed rumen samples, which is mostly attributed to members of the genus Ca. Limivicinus (*Oscillospiraceae*) (Fig. S4, Tables S6 and S9), we identified only a few 16S rRNA gene- and *yihQ* ASVs/OTUs that were significantly enriched in SQ-amended microcosms compared to unamended controls (Tables S10 and S11). 16S rRNA gene-ASV 1 and 2 were related to the genera *Caproiciproducens* (*Acutalibacteraceae*) and Ca. Mailhella (*Desulfovibrionaceae*) and reached a maximal relative abundance of ~2.7% and 0.7% in SQ-amended microcosms, respectively (Fig. 3D and E). Additionally, seven *yihQ* ASVs that clustered in two OTUs were significantly enriched in SQ-amended microcosms. OTU 1 (ASVs 2–7), classified as Ca. Limivicinus (*Oscillospiraceae*), and OTU 2 (ASV 1), classified as an unknown *Sphaerochaetaceae* bacterium, reached a maximal relative *yihQ* abundance of 12.6% and 9% in SQ-amended microcosms, respectively (Fig. 3F and G, Fig. S7, Table S11).

**Figure 4:**
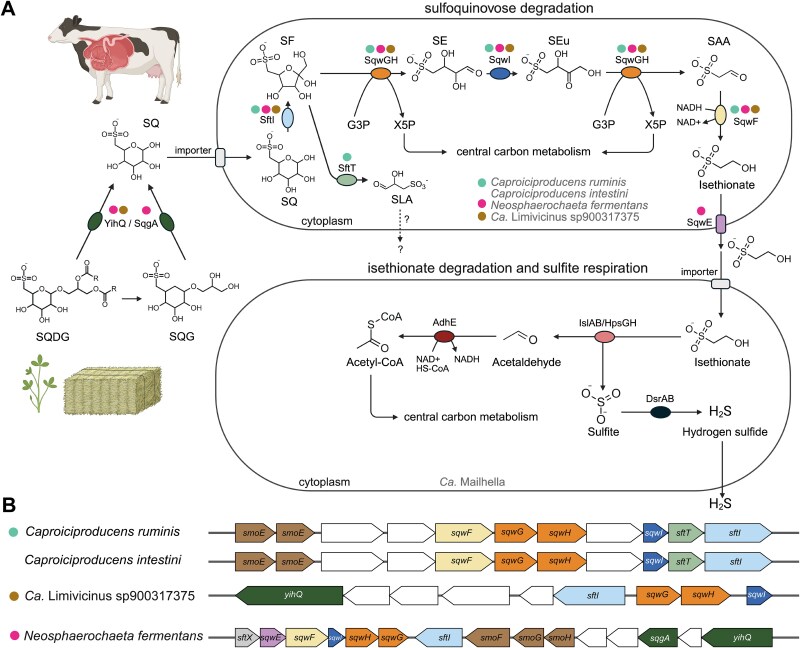
Sulfoquinovose may be cooperatively degraded to sulfide via isethionate cross-feeding from diverse sulfo-transketolase-pathway-encoding *Clostridia* species to organosulfur-respiration-encoding Ca. *Mailhella* species. (A) Cows may ingest sulfoquinovosyl diacylglycerol (SQDG) through hay, grass, and dietary algae supplements. Newly described bacteria from the cow rumen have the capacity for sulfoquinovose (SQ) catabolism. The sulfoquinovosidases YihQ or SqgA initially cleave SQ from sulfoquinovosyl diacylglycerol (SQDG) or sulfoquinovosyl glycerol (SQG). SQ is further catabolized to isethionate by the sulfo-transketolase pathway-encoding rumen species *Caproiciproducens ruminis* MAG SQrumen1, *Caproiciproducens intestini* MAG SQrumen2, *Neosphaerochaeta fermentans* MAG SQrumen3, and Ca. Limivicinius sp900317375 (GCA_900317375). Both *Caproiciproducens* MAGs additionally encode 6-deoxy-6-sulfofructose transaldolase SftT that produces 3-sulfolactaldehyde (SLA). A Ca. *Mailhella* species may desulfonate isethionate to acetaldehyde and sulfite by the isethionate sulfite-lyase (IslAB) and/or DHPS sulfite-lyase complexes (HpsGH). Sulfite is used as an electron acceptor for anaerobic respiration via the DsrAB-dissimilatory sulfite reductase system. The figure was created with biorender.com. Coloured dots above enzymes indicate presence and transcription of the respective gene in *Caproiciproducens ruminis* MAG SQrumen1 and *Caproiciproducens intestini* MAG SQrumen2 (turquoise), *Neosphaerochaeta fermentans* MAG SQrumen3 (pink), and Ca. *Limivicinus* sp900317375 (brown). Functionally equivalent enzymes are grouped under a single name based on the first characterized homolog (e.g. SftI corresponds to SqvD). (B) Gene clusters for SQ degradation via the sulfo-transketolase pathway in *Caproiciproducens intestini, Caproiciproducens ruminis, Neosphaerochaeta fermentans* and Ca. *Limivicinus* sp900317375 (GCA_900317375). Genes depicted in white have no assigned function in sulfur metabolism. YihQ and SqgA, sulfoquinovosidase; SftI, SQ isomerase; SqwGH, 6-deoxy-6-sulfofructose transketolase, SqwI, 4-deoxy-4-sulfoerythrose isomerase; SqwF, sulfoacetaldehyde reductase; SqwE, isethionate exporter; IslAB, isethionate sulfite-lyase; HpsGH, DHPS sulfite-lyase; DsrAB, dissimilatory sulfite reductase; AdhE, aldehyde-alcohol dehydrogenase; SmoE, sulfoquinovosyl glycerol transport ATP-binding protein; SftT, 6-deoxy-6-sulfofructose transaldolase; SmoFGH, transporter; SftX, DUF4867. SQ, sulfoquinovose; SF, 6-deoxy-6-sulfofructose; SE, 4-deoxy-4-sulfoerythrose; SEu, 4-deoxy-4-sulfoerythrulose; G3P, glycerol-3-phosphate; X5P, xylulose-5-phosphate; SAA, sulfoacetaldehyde; DHPS, 2,3-dihydroxypropane-1-sulfonate.

Genome-centric metagenomics recovered 25 medium- to high-quality MAGs from the SQ-amended microcosms, including *Caproiciproducens*, Ca. Limivicinus, *Sphaerochaetaceae*, and Ca. Mailhella MAGs (Table S12). The latter MAGs represent yet uncultivated and undescribed species and/or genera. *Caproiciproducens* MAGs SQrumen1 and SQrumen2 each represent a different species (93.3% ANI) [41]. *Caproiciproducens* MAG SQrumen2 belongs to the GTDB species *Caproiciproducens* sp000752215 (GCA_000752215) (97.1% ANI), whereas *Caproiciproducens* MAG SQrumen1 (93.3% ANI to *Caproiciproducens* sp000752215, GCA_000752215) represents a yet unknown species. The two Ca. Limivicinus MAGs SQrumen19 and SQrumen23 are only ~50% complete and were thus not used for ANI/AAI calculations. Ca. Limivicinus MAG SQrumen19 was classified as the GTDB species Ca. Limivicinus sp900317375 (GCA_900317375), while Ca. Limivicinus MAG SQrumen23 may represent a yet unknown species (Table S12). *Sphaerochaetaceae* MAG SQrumen3 belongs to the GTDB species JAAYFO01 sp028691695 (GCA_028691695) (99% ANI) and has <53% AAI to species outside the GTDB genus JAAYFO01, suggesting it represents a yet undescribed genus [55]. Ca. Mailhella MAG SQrumen6 belongs to the GTDB species Ca. Mailhella sp902783285 (GCA_902783285) (98.8% ANI). Consistent with the metabolite data, the *Caproiciproducens* and *Sphaerochaetaceae* MAGs and Ca. Limivicinus sp900317375 (GCA_900317375) encode the sulfo-transketolase pathway variant for SQ degradation that produces isethionate (Fig. 4A) [14]. Furthermore, genome-based metatranscriptomics of rumen microcosms during SQ degradation showed that transcription levels of the core sulfo-transketolase pathway genes (*sqwG, sqwH, sqwI*) consistently ranked among the top 15% of the genes in each enriched species (Fig. S8, Tables S13–S17). A more comprehensive genome analysis revealed that 10.7% of 28 *Caproiciproducens*, 35.5% of 313 Ca. Limivicinus (4.7% of 3230 *Oscillospiraceae*), and 11.8% of 246 *Sphaerochaetaceae* genomes in the GlobDB database have the capacity for SQ degradation via the sulfo-transketolase pathway. The three previously available genomes of the *Sphaerochaetaceae* genus JAAYFO01 do not encode the sulfo-transketolase pathway (Table S18). All sulfo-transketolase pathway-encoding *Caproiciproducens* genomes lacked genes for the two known sulfoquinovosidases YihQ and SqgA [23] (Fig. 4B). This explains the absence of enriched *yihQ* ASVs of this genus in the SQ-supplemented rumen microcosms (Table S11) and suggests that SQ-degrading *Caproiciproducens* species either rely on other microorganisms for SQ release or harbor a yet unknown sulfoquinovosidase. Sulfo-transketolase pathway-encoding *Caproiciproducens* species additionally contained and transcribed 6-deoxy-6-sulfo-D-fructose transaldolase-encoding *sftT* at high levels (Figs. 4 and S8, Tables S13–S14). However, they lacked genes for the 3-sulfolactaldehyde reductase (*sftR*) and dehydrogenase (*sftD*), indicating an incomplete sulfo-transaldolase pathway, which is in line with absence of DHPS and 3-sulfolactate in the microcosms [30].

Our amplicon sequencing approach focused on *yihQ* and therefore may have underestimated the richness and activity of SQ-degrading cow rumen microorganisms that employ the NAD^+^-dependent sulfoquinovosidase SqgA. To address this limitation, we analyzed presence of *yihQ* and *sqgA* in our metagenomes from the SQ-amended rumen microcosms (120 h time point) and in a public collection of 5578 microbial genomes from the cow rumen [56]. The ratio of the number of metagenome reads of *yihQ* and *sqgA*, corrected by the median gene length, was 2.1:1 (Table S19). Furthermore, in the public rumen genome collection, 197 genomes encoded YihQ, whereas only six genomes encoded SqgA, and only two genomes encoded both YihQ and SqgA (Table S20). Genomes encoding YihQ were predominantly affiliated with the families *Acutalibacteraceae* (n = 65), *Oscillospiraceae* (n = 48), and *Lachnospiraceae* (n = 31), but also the *Sphaerochaetaceae* (n = 5). Overall, these results suggest that most SQ-metabolizing bacteria in the cow rumen rely on YihQ sulfoquinovosidases for the release of SQ from SQDG or SQG.

**Figure 5:**
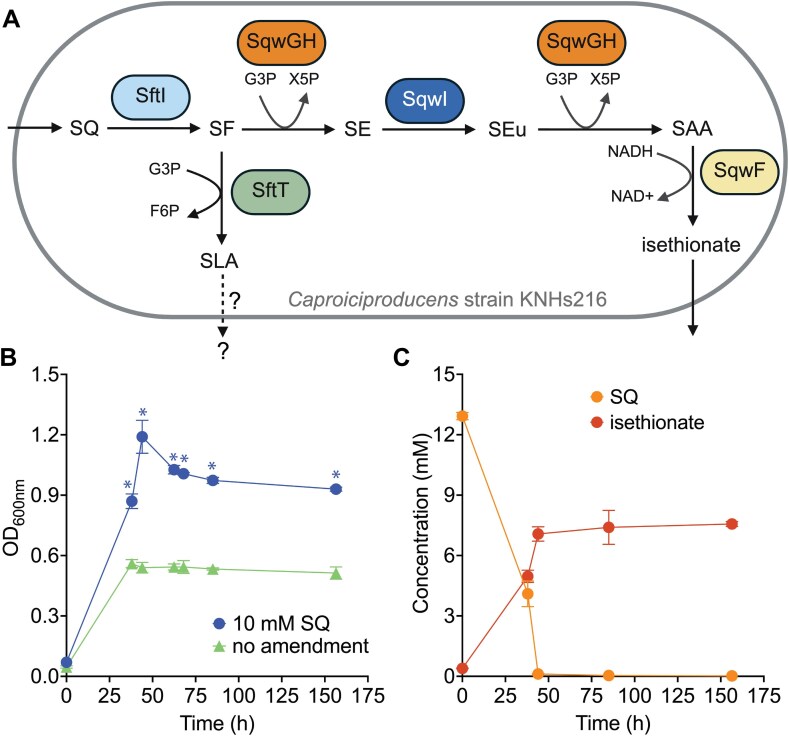
Sulfoquinovose metabolism of *Caproiciproducens* strain KNHs216. Schematic overview of genome-predicted sulfoquinovose (SQ) degradation via the bifurcated sulfo-transketolase/transaldolase pathway in *Caproiciproducens* strain KNHs216 (created with biorender.com) (A). SQ is converted to 6-deoxy-6-sulfofructose (SF) by the SQ isomerase SftI (gene locus tag LY85_RS15350). SF is then further metabolized either to 3-sulfolactaldehyde (SLA) by the 6-deoxy-6-sulfofructose transaldolase SftT (gene locus tag LY85_RS15345) or to isethionate via the sulfo-transketolase pathway. The question marks indicate that genes for the 3-sulfolactaldehyde reductase (sftR) and dehydrogenase (sftD) [13, 30] are absent. This suggests that the sulfo-transaldolase pathway is either incomplete or SLA is further metabolized by currently unknown enzymes. In the sulfo-transketolase pathway, the 6-deoxy-6-sulfofructose transketolase SqwGH (gene locus tags LY85_RS15325 and LY85_RS15330) transfers two consecutive C_2_-moieties onto glyceraldehyde-3-phosphate (G3P) resulting in two molecules of xylulose-5-phosphate (X5P) for the central carbon metabolism. After the first transketolation, 4-deoxy-4-sulfoerythrose (SE) is isomerized to 4-deoxy-4-sulfoerythrulose (SEu) by the 4-deoxy-4-sulfoerythrose isomerase SqwI (gene locus tag LY85_RS15340). After the second transketolation, sulfoacetaldehyde (SAA) is reduced to isethionate via the sulfoacetaldehyde reductase SqwF (gene locus tag LY85_RS15320). Anoxic incubations of *Caproiciproducens* strain KNHs216 were amended with 10 mM SQ (n = 3) or left unamended (n = 3). Cultures amended with SQ reached a significantly higher optical density at 600 nm (OD_600nm_) than unamended cultures (B). SQ (orange) degradation coincided with the production of ~7 mM isethionate (red) (C). In B and C, lines show averages of triplicate incubations with error bars representing one standard deviation. Asterisks show significant differences (ANOVA with Bonferroni post hoc test; adjusted *P* values <0.05) between SQ-amended incubations and controls without SQ. F6P, fructose-6-phosphate.

We additionally performed anaerobic growth tests with *Caproiciproducens* strain KNHs216 to confirm SQ degradation by a member of the genus *Caproiciproducens*. Strain KNHs21 represents a different *Caproiciproducens* species (GTDB species *Caproiciproducens* sp000752215) but encodes the same bifurcated sulfo-transketolase/transaldolase pathway genes as the two rumen MAGs (Fig. 5A). Rich medium cultures supplemented with SQ showed significantly higher growth yields than cultures without SQ (Fig. 5B, Fig. S9). Complete removal of SQ led to isethionate production and coincided with onset of the stationary growth phase of strain KNHs216 (Fig. 5C). The stoichiometric difference in the conversion of SQ to isethionate is possibly due to the metabolic partitioning of 6-deoxy-6-sulfo-D-fructose into the isethionate-producing transketolase path and the transaldolase path, the latter producing a currently unidentified sulfonated product in *Caproiciproducens* (Figs. 4A and 5A).

The sulfo-transaldolase and sulfo-Embden-Meyer-Parnas pathways, which are primarily used for SQ fermentation by gut bacteria of humans and laboratory mice [24, 25], employ three carbon atoms of SQ for central carbon metabolism and excrete a C_3_-sulfonate (DHPS, 3-sulfolactate) [10, 13, 14]. In comparison, the sulfo-transketolase pathway of the identified cow rumen bacteria utilizes four SQ carbon atoms for the central carbon metabolism, excretes a C_2_-sulfonate (isethionate), and may thus maximize the energy yield compared to other SQ fermentation pathways [14, 57].

The slightly increased sulfide production in SQ-amended microcosms likely resulted from anaerobic respiration of isethionate by a Ca. Mailhella species (Figs. 3C and 4A). The genus Ca. Mailhella forms a monophyletic group with the genera *Bilophila* and *Taurinivorans*, which comprise important organosulfonate-respiring bacteria from the human and mouse gut, respectively [58]. Ca. Mailhella MAG SQrumen6 and 75% of 48 Ca. Mailhella genomes harbour genes that are homologous to the paralogous genes for DHPS sulfite-lyase (*hpsGH*) and isethionate sulfite-lyase (*isIAB*) complexes of *Bilophila wadsworthia* [15, 24, 59]. Both HpsG and IslA can cleave isethionate to sulfite and acetaldehyde, yet HpsG shows higher activity for DHPS desulfonation [15, 59]. Sulfite respiration to sulfide is catalyzed by the dissimilatory sulfite reductase *dsrAB* system, which is evolutionary conserved in *Desulfobacterota* members, including Ca. Mailhella (Table S18) [58, 60]. Consistent with their proposed ecophysiological role, transcription levels of core genes for isethionate-based respiration of sulfite to sulfide in the SQ-amended microcosms were among the top 26% of the genes in Ca. Mailhella MAG SQrumen6 (Fig. S8, Table S17).

In summary, our results indicate that diverse, previously undescribed bacterial species in the rumen cooperatively mineralize plant-derived SQ to hydrogen sulfide through isethionate-cross-feeding. These findings carry implications for animal husbandry, as increased sulfite respiration has been associated with decreased activity of methanogenic archaea [61]. Various micro- and macroalgae are tested as feed additives to reduce emissions of the greenhouse gas methane from ruminants [62, 63]. Future research may investigate whether SQDG present in algae contributes to their anti-methanogenic properties [64].

### Proposal of new taxa

In accordance with the SeqCode [65], we propose the following new taxa for three high-quality MAGs (Fig. 4): *Caproiciproducens ruminis* sp. nov. MAG SQrumen1, *Caproiciproducens intestini* sp. nov. MAG SQrumen2, and *Neosphaerochaeta fermentans* gen. nov., sp. nov. MAG SQrumen3.

### Description of *Caproiciproducens ruminis* sp. nov.

*Caproiciproducens ruminis* sp. nov. (ru’mi.nis. L. gen. neut. n. *ruminis*, of the rumen, the source of the bacterium). The designated DNA sequence is MAG SQrumen1 recovered from cow rumen. This species was enriched in anoxic incubations of rumen fluid with sulfoquinovose. Genome-centric metatranscriptomic analysis suggests that sulfoquinovose is fermented via a bifurcated sulfo-transketolase/transaldolase pathway to isethionate and an unidentified sulfonate product. The NCBI accession number for the genome is SAMN48198398.

### Description of Caproiciproducens intestini sp. nov.

*Caproiciproducens intestini* sp. nov. (in.tes.ti’ni. L. gen. neut. n. *intestini*, of the gut, referring to the origin of the bacterium). The designated DNA sequence is MAG SQrumen2 recovered from cow rumen. This species was enriched in anoxic incubations of rumen fluid with sulfoquinovose. Genome-centric metatranscriptomic analysis suggests that sulfoquinovose is fermented via a bifurcated sulfo-transketolase/transaldolase pathway to isethionate and an unidentified sulfonate product. The species appears to have a broader ecological distribution beyond the intestinal tract, with representation in landfill, wastewater, manure, and anaerobic fermenter metagenomes [66, 67]. The NCBI accession number for the genome is SAMN48198399.

### Description of Neosphaerochaeta fermentans sp. nov.

*Neosphaerochaeta fermentans* sp. nov. (fer.men’tans. L. part. adj. *fermentans*, fermenting) The designated DNA sequence is MAG SQrumen3 recovered from cow rumen. This species was enriched in anoxic incubations of rumen fluid with sulfoquinovose. Genome-centric metatranscriptomic analysis suggests sulfoquinovose is fermented to isethionate via the sulfo-transketolase pathway. The genome additionally encodes two different sulfoquinovosidases (YihQ and SqgA). The species appears to have a broader ecological distribution beyond the intestinal tract, with representation in aquatic, bioreactor, and soil metagenomes [66, 67]. The NCBI accession number for the genome is SAMN48198401.

### Description of *Neosphaerochaeta* gen. nov.

*Neosphaerochaeta* gen. nov. (ne.o.sphae.ro.chae’ta. Gr. masc. adj. neos, new; N.L. fem. n. *Sphaerochaeta*, a bacterial genus; N.L. fem. n. *Neosphaerochaeta*, a new genus related to *Sphaerochaeta*). Type species: *Neosphaerochaeta fermentans* sp. nov., family: *Sphaerochaetaceae*.

## Conclusions

We developed functional gene amplicon sequencing assays for analyzing the richness of SQDG/SQ-degrading microorganisms in complex samples. The two PCR assays both target the same gene region of the YihQ sulfoquinovosidase and exhibited similar environment-dependent specificity. They were highly specific for *yihQ* in intestinal samples from humans, mice, and cows but amplified a substantial proportion of non-*yihQ* sequences from a marine sediment sample. To ensure accurate *yihQ* richness analysis, we implemented a computational workflow to efficiently remove non-target sequences. However, the potential amplification of non-target genes may reduce the sensitivity of the PCR assays in certain environments, such as marine sediments. Future improvements to the *yihQ*-targeted PCR assays could address these limitations. Additionally, for a more comprehensive assessment of SQDG/SQ-degrading microorganisms, functional gene amplicon sequencing should be complemented by PCR assays targeting the more recently identified SqgA sulfoquinovosidase [23].

Among all analyzed samples, rumen fluid exhibited the highest *yihQ* richness, suggesting that the species/strain-level diversity of potential SQDG/SQ-degraders in the cow gut is one to two orders of magnitude greater than in the human and mouse gut. Furthermore, yet uncultured species were enriched in rumen fluid microcosms supplemented with SQ and identified as primary (*Caproiciproducens*, Ca. Limivicinus, *Neosphaerochaeta*) or secondary (Ca. Mailhella) SQDG/SQ-degraders through metabolite analysis, amplicon sequencing, and genome-resolved metagenomics and metatranscriptomics. This study revealed SQDG/SQ degradation as a fundamental metabolic trait of the cow rumen microbiome and provided insights into the diversity and metabolic potential of the newly described taxa involved.

## Supplementary Material

wrag069_Supplemental_Files

## Data Availability

The sequence datasets generated during the current study are available in the NCBI repository under BioProject ID PRJNA1242727 (accession numbers of amplicon data: SRX28160950-SRX28160955, SRX28160963-SRX28160974, SRX28166061-SRX28166142, metagenome data: SRR33333717-SRR33333719, metatranscriptome data: SRX30952554-SRX30952559, MAGs: SAMN48198398-SAMN48198401; SAMN48372048-SAMN48372068). The YihQ reference database, the amino acid alignment of YihQ reference sequences, the YihQ reference tree, and further information are available in Additional Files 1–7. HMMs for microbial organosulfur metabolism proteins are available at github.com/TSTanabe/HMSS2/tree/main/Hidden_Markov_Models.
